# Advanced atomic force microscopy techniques II

**DOI:** 10.3762/bjnano.5.241

**Published:** 2014-12-03

**Authors:** Thilo Glatzel, Ricardo Garcia, Thomas Schimmel

**Affiliations:** 1 Department of Physics, University of Basel, Klingelbergstrasse 82, 4056 Basel, Switzerland; 2 Instituto de Microelectronica de Madrid, CSIC Isaac Newton 8, 28760 Tres Cantos, Madrid, Spain; 3 Institute of Nanotechnology (INT), Karlsruhe Institute of Technology (KIT), 76021 Karlsruhe, Germany

**Keywords:** AFM

Surface science and nanotechnology are inherently coupled because of the increased surface-to-volume ratio at the nanometer scale. Most of the exciting and astonishing properties of nanoscale materials are related to certain surface reconstructions and nanoscale geometries. New functionality is achieved by combinations of nanoscale materials or by structuring their surfaces. The unrivaled tools for measurements of all kind of nanoscale properties are scanning probe microscopy (SPM) techniques, which were triggered by the invention of the scanning tunneling microscope (STM) in 1982 [1–3] and of the atomic force microscope (AFM) in 1986 [4]. These tools opened a huge field of nanoscale studies, from metal surfaces and clusters, molecular structures, insulators to liquid and electrochemical environments and even allowed the integration of various SPM techniques into biological and chemical experiments.

The second volume of the Thematic Series “Advanced atomic force microscopy techniques”, which is presented here, compiles again exciting developments in nanoscale research based on SPM techniques addressed by the scientific community within the last years. Similar to the first volume [5], the development of advanced techniques and their application is the focus of this Thematic Series. Contributions related to energy conversion and storage systems have been addressed, e.g., the analysis of cathodes of lithium–sulfur batteries for a comparison of their nanoscale electrical, electrochemical, and morphological properties [6] or the analysis of CdS quantum dots on TiO_2_ by a combination of AFM and X-ray photoelectron spectroscopy [7]. The folding and rupture of graphene on SiC analyzed by non-contact AFM and Kelvin probe force microscopy [8] are presented as well as molecular structures such as the self-assembly of multidentate organothiols onto Au(111), which were studied in situ by using scanning probe nanolithography and time-lapse AFM [9]. Patterns of thiol-based self-assembled monolayers for the site-selective growth of metal-organic frameworks have been created and analyzed by a nanografting technique by using an AFM as a structuring tool [10]. The effect of Cu intercalation at the interface of self-assembled monolayers and a Au(111)/mica substrate was analyzed by STM [11] as well as the growth behavior of PTCDA islands [12].

Furthermore, the analysis of mechanical properties of either nanoparticles [13] or biological systems [14–16] is covered by several articles and reviewed by Cohen and co-workers [17]. Especially the application of advanced SPM techniques in biology provides exciting new results and clearly shows a route for development for the next years. All of the new applications and experiments are strongly dependent on theoretical and technical developments. Virtual AFMs used to simulate AFM measurements [18–20] and to deconvolute complex correlations between various surface properties [21] are based on the implementation of proportional-integral controllers to give realistic feedback behaviours. Stirling proposed a theoretical model for studying the SPM feedback in the context of control theory providing the possibility to understand and model the performance from SPM systems with real parameters [22]. Furthermore, technical contributions discuss the impact of thermal frequency drift of quartz-based force sensors at low temperatures to the accuracy of the force measurements [23] and the trade-offs in sensitivity and sampling depth in bimodal and trimodal AFM [24]. The examples mentioned give a first impression of this collection of high quality research provided to the Beilstein Journal of Nanotechnology, the open-access journal for publication and dissemination of nanoscience research results. We are convinced that the articles presented here will stimulate new ideas in the research field.

We would like to thank all of the authors for their excellent contributions and the referees for their comprehensive and valuable reports, sustaining a journal that is attractive for contributors. Finally, we would also like to thank the team at the Beilstein-Institut for their excellent support and acknowledge the open-access policy of the Beilstein Journal of Nanotechnology, which provides the professional framework and support allowing the collection, review, publishing, and distribution of research results in an easy and excellent way.

Thilo Glatzel, Ricardo Garcia & Thomas Schimmel

November 2014

## References

[1] Binnig G, Rohrer H, Gerber C, Weibel E (1982). Phys Rev Lett.

[2] Binnig G, Rohrer H, Gerber C, Weibel E (1982). Appl Phys Lett.

[3] Binnig G, Rohrer H (1982). Helv Phys Acta.

[4] Binnig G, Quate C F, Gerber C (1986). Phys Rev Lett.

[5] Glatzel T, Hölscher H, Schimmel T, Baykara M Z, Schwarz U D, Garcia R (2012). Beilstein J Nanotechnol.

[6] Hiesgen R, Sörgel S, Costa R, Carlé L, Galm I, Cañas N, Pascucci B, Friedrich K A (2013). Beilstein J Nanotechnol.

[7] Ghazzal M N, Wojcieszak R, Raj G, Gaigneaux E M (2014). Beilstein J Nanotechnol.

[8] Temmen M, Ochedowski O, Kleine Bussmann B, Schleberger M, Reichling M, Bollmann T R J (2013). Beilstein J Nanotechnol.

[9] Tian T, Singhana B, Englade-Franklin L E, Zhai X, Lee T R, Garno J C (2014). Beilstein J Nanotechnol.

[10] Ladnorg T, Welle A, Heissler S, Wöll C, Gliemann H (2013). Beilstein J Nanotechnol.

[11] Shen C, Buck M (2014). Beilstein J Nanotechnol.

[12] Zebari A A A, Kolmer M, Prauzner-Bechcicki J S (2013). Beilstein J Nanotechnol.

[13] Maharaj D, Bhushan B (2014). Beilstein J Nanotechnol.

[14] Guzman H V, Garcia R (2013). Beilstein J Nanotechnol.

[15] Tatlybaeva E B, Nikiyan H N, Vasilchenko A S, Deryabin D G (2013). Beilstein J Nanotechnol.

[16] Ramos J R, Pabijan J, Garcia R, Lekka M (2014). Beilstein J Nanotechnol.

[17] Cohen S R, Kalfon-Cohen E (2013). Beilstein J Nanotechnol.

[18] Nony L, Baratoff A, Schär D, Pfeiffer O, Wetzel A, Meyer E (2006). Phys Rev B.

[19] Polesel-Maris J, Gauthier S (2005). J Appl Phys.

[20] Canova F F, Foster A S (2011). Nanotechnology.

[21] Elias G, Glatzel T, Meyer E, Schwarzman A, Boag A, Rosenwaks Y (2011). Beilstein J Nanotechnol.

[22] Stirling J (2014). Beilstein J Nanotechnol.

[23] Pielmeier F, Meuer D, Schmid D, Strunk C, Giessibl F J (2014). Beilstein J Nanotechnol.

[24] Eslami B, Ebeling D, Solares S D (2014). Beilstein J Nanotechnol.

